# FBXL6 is a vulnerability in AML and unmasks proteolytic cleavage as a major experimental pitfall in myeloid cells

**DOI:** 10.1038/s41375-024-02345-0

**Published:** 2024-07-16

**Authors:** Anna Sperk, Antje Gabriel, Daniela Koch, Abirami Augsburger, Victoria Sanchez, David Brockelt, Rupert Öllinger, Thomas Engleitner, Piero Giansanti, Romina Ludwig, Priska Auf der Maur, Wencke Walter, Torsten Haferlach, Irmela Jeremias, Roland Rad, Barbara Steigenberger, Bernhard Kuster, Ruth Eichner, Florian Bassermann

**Affiliations:** 1 Department of Medicine III, Klinikum rechts der Isar, Technical University of Munich, Munich, Germany; 2 TranslaTUM, Center for Translational Cancer Research, Technical University of Munich, Munich, Germany; 3 Mass Spectrometry Core Facility, Max Planck Institute of Biochemistry, Martinsried, Germany; 4 Medical Department II, Translational Gastroenterological Oncology, Klinikum rechts der Isar, Technical University of Munich, Munich, Germany; 5 Institute of Molecular Oncology and Functional Genomics, Technical University of Munich, Munich, Germany; 6 Bavarian Center for Biomolecular Mass Spectrometry at the University Hospital rechts der Isar BayBioMS@MRI, Technical University of Munich, Munich, Germany; 7 Research Unit Apoptosis in Hematopoietic Stem Cells, Helmholtz Center Munich, Munich, Germany; 8 MLL Munich Leukemia Laboratory, Munich, Germany; 9 Department of Pediatrics, Dr. Von Hauner Children’s Hospital, LMU University Hospital, LMU Munich, Munich, Germany; 10 Proteomics and Bioanalytics, School of Life Sciences, Technical University of Munich, Freising, Germany; 11 Deutsches Konsortium fürr Translationale Krebsforschung (DKTK), Heidelberg, Germany; 12 Bavarian Cancer Research Center (BZKF), Munich, Germany

**Keywords:** Cell biology, Acute myeloid leukaemia

## To the Editor:

Acute myeloid leukemia (AML) is a heterogeneous blood-borne malignancy with an overall 5-year survival rate of 25 percent [1]. Despite substantial genetic characterization in the last decades, only a few targeted therapies, such as FLT3 inhibitors, have entered clinical practice. Treatment still largely relies on chemotherapy and hematopoietic stem cell transplantation in younger patients, and epigenetic therapies as azacytidine in combination with the Bcl-2 inhibitor venetoclax in elderly patients, with the first causing relatively high rates of therapy-associated morbidity and mortality, and the latter showing limited responses [2]. Therefore, the identification of new actionable vulnerabilities, beyond genetics and epigenetics, is highly demanded. Approaches investigating aberrant mechanisms on protein level, such as disease-specific post-translational modifications (PTM), hold great promise to provide new therapeutic targets. Ubiquitylation marks the major PTM that regulates the abundance of proteins, thus orchestrating key processes as proliferation, survival, and differentiation [3, 4]. SCF (SKP1/CUL1/F-box) E3 ubiquitin ligases are characterized by modular complexes containing one of the 72 individual F-box proteins as substrate recruiting receptors, the F-box adapter SKP1, the scaffold protein CUL1, and the RING protein RBX1 [5]. While most SCF complexes have not yet been linked to substrates or biological activities, individual members have been found to play key roles in different tumor entities [6, 7]. Recent reports have now provided first insights into the roles of SCF E3 ligases in AML. Next to identifying the F-box protein FBXL2 as a tumor suppressor [8], the NEDD8 inhibitor pevonedistat showed some additional benefit when combined with azacytidine in AML patients in clinical trials [9], and is undergoing further investigation in AML and further hematological malignancies. Notably, NEDD8 inhibitors prevent the neddylation-dependent activation of all cullin-based RING E3 ubiquitin ligases (CRLs), which include SCF complexes. We therefore hypothesized that some SCF ubiquitin ligases, or their substrate-recruiting F-box proteins, are specifically deregulated in AML, thus representing potential therapeutic targets.

**Fig. 1:**
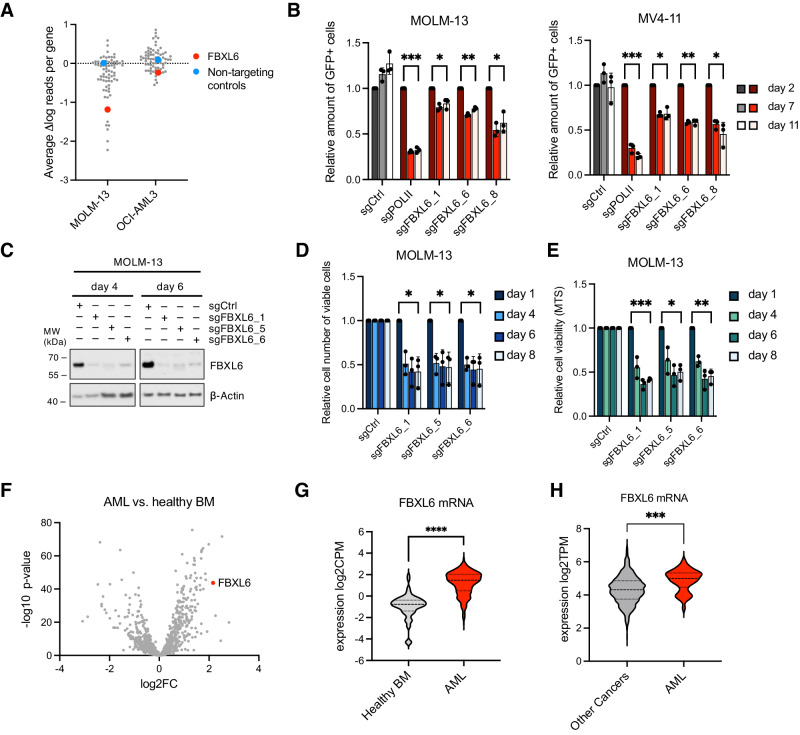
CRISPR/Cas9-based screening identifies FBXL6 as a novel vulnerability in AML. **A** Results from CRISPR/Cas9 drop-out screens using a custom-designed sgRNA knockout library targeting all 72 human F-box proteins in 2 AML cell lines. The delta of normalized and log transformed sgRNA read‐counts of day 14 and day 0 was calculated and scores of all sgRNAs targeting the same gene or controls were averaged and are shown as one data point. **B** Competitive growth assay of Cas9-expressing AML cell lines transduced with GFP-expressing sgRNA constructs targeting *FBXL6* (sgFBXL6), *POLII* (sgPOLII) as positive control or non-targeting control (sgCtrl) at 30–50% efficiency. The ratio of GFP positive to non-transduced cells was measured by flow cytometry on the indicated days and normalized to day 2. ***P < 0.001; **P < 0.01; *P < 0.05, by One sample t-test. **C** Immunoblot analysis of AML Cas9 cell lines transduced with sgRNA constructs targeting *FBXL6* or sgCtrl. Cells were harvested at the indicated time points post-infection and whole-cell extracts prepared under denaturing lysis conditions. **D** Cells from **C** and two additional biological replicates were counted using the trypan blue exclusion method on an automatic cell counter (Vi-Cell Blu, Beckman Coulter) at the indicated time points. **E** Cells from **C** and two additional biological replicates were subjected to MTS viability assays using the CellTiter 96® Aqueous One Solution at the indicated time points. Results in **D** and **E** are normalized and presented in relation to sgCtrl. ***P < 0.001; **P < 0.01 ; *P < 0.05, by One sample t-test. **F** Differential gene expression analysis of 1041 Ubiquitin (Ub)-related genes in the MLL AML patient cohort (*n* = 762) compared to healthy bone marrow (BM) controls (*n* = 64). Log2FC (fold change) and the associated *p*-value are shown. **G** Individual values from **F** for *FBXL6* mRNA expression in AML patients and healthy BM. CPM counts per million reads mapped. ****P < 0.0001, by Student’s *t* test. **H** *FBXL6* mRNA expression across AML and other cancer cell lines derived from the DepMap public 23Q4 dataset. TPM transcripts per million reads mapped. ***P < 0.001 by Student’s *t* test.

In order to screen for AML-specific vulnerabilities within the family of SCF complexes, we first generated a focused CRISPR/Cas9-based knockout library covering all 72 human genes coding for F-box proteins (sgRNA sequences derived from the genome-scale CRISPR Knock-Out library GeCKOv2 [10]), together with positive and negative controls. Using this custom library, we performed pooled CRISPR/Cas9 drop-out screens in two independent AML cell lines and identified the poorly studied F-box protein FBXL6 as the most prominent common dependency between OCI-AML3 (*DNMT3A* and *NMP1* mutated) and MOLM-13 (*FLT3-ITD*) cells (Fig. 1A, Supplementary Table 1). Confirmatory assays indeed demonstrated a significant decrease in proliferation upon knockout of *FBXL6* in AML cell lines as determined by competitive growth experiments, trypan blue exclusion cell counting and MTS assays (Fig. 1B–E, Supplementary Fig. 1A). Notably, the effects of FBXL6 loss were stronger in the two *FLT3-ITD* mutated AML cell lines MOLM-13 and MV4-11 as compared to the *DNMT3A* and *NMP1* mutated OCI-AML3 line (Fig. 1B–E, Supplementary Fig. 1A), potentially pointing towards a more specific dependency.

To cross validate FBXL6 as a potential dependency in AML, we analyzed RNAseq data of a large AML patient cohort encompassing more than 700 cases [11]. Within the over 1,000 ubiquitin-related genes investigated, *FBXL6* was one of most highly overexpressed genes (Fig. 1F). Importantly, across all AML subtypes according to the WHO classification, over 90% of AML cases displayed higher *FBXL6* mRNA levels than healthy control bone marrow (Fig. 1G, Supplementary Fig. 1B). Likewise, analysis of the RNAseq Expression Public 23Q4 dataset from DepMap, covering approximately 1400 different cancer cell lines, revealed particularly high *FBXL6* mRNA expression levels in AML cell lines as compared to other cancer cell lines (Fig. 1H). Moreover, analysis of the BEAT AML 2.0 cohort [12] revealed high FBXL6 mRNA expression levels to correlate with adverse prognosis according to the ELN guidelines [2] in further support of an oncogenic role (Supplementary Fig. 1C).

**Fig. 2:**
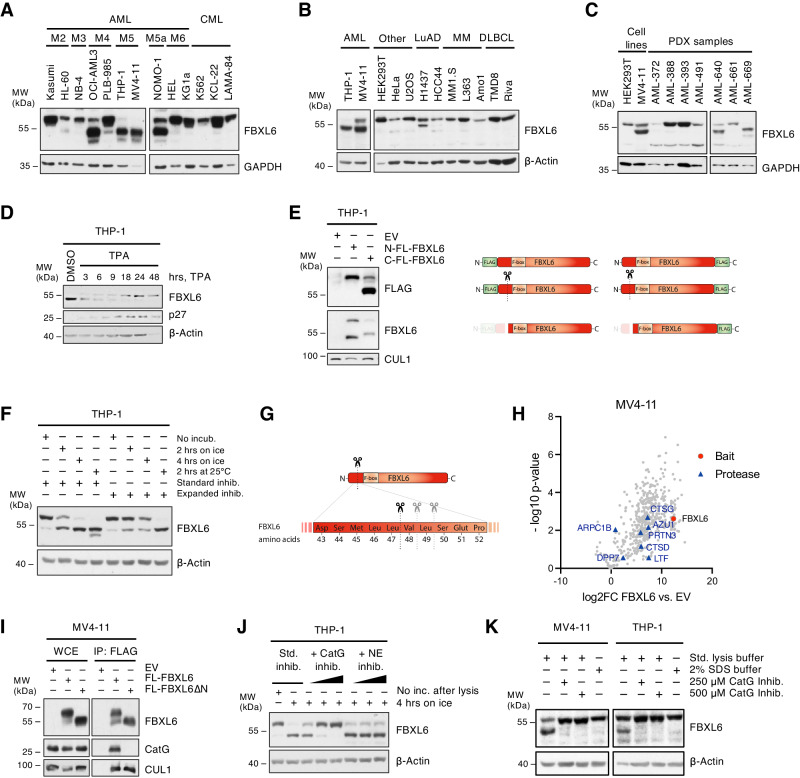
FBXL6 underlies excessive protease cleavage by CatG in cell lysates. Immunoblot analyses of whole-cell extracts (WCE) prepared under standard lysis conditions from cell lines of (**A**) different AML subtypes and selected CML (chronic myeloid leukemia) cell lines, and (**B**) various other cancer entities including lung adenocarcinoma (LuAD), multiple myeloma (MM) and diffuse large B-cell lymphoma (DLBCL). **C** Immunoblot analyses of whole-cell extracts (WCE) prepared under standard lysis conditions from cell lines and different AML patient-derived xenograft lines. **D** Immunoblot analysis of THP-1 cells treated with 25 ng/ml TPA or DMSO control for the indicated timepoints. **E** Immunoblot analysis of THP-1 cells lentivirally transduced to ectopically express N- or C-terminally FLAG-tagged FBXL6 (N-FL-FBXL6, C-FL-FBXL6) or empty vector (EV) control. WCE of infected cells were prepared under standard lysis conditions. Right side: scheme to visualize the tagged forms and resulting cleavage fragments of FBXL6. **F** In-vitro-cleavage assay using THP-1 lysates which were incubated on ice or at 25 °C for the indicated periods of time or denatured directly by addition of Laemmli buffer. Standard inhibitor cocktail contains aprotinin, leupeptin, soybean trypsin inhibitor, PMSF, TPCK, TLCK; expanded inhibitor cocktail additionally comprises AEBSF, bestatin, E-64, and pepstatin. **G** Scheme of identified cleavage sites in the N-terminal region of FBXL6 (see also Supplementary Fig. 4) **H** Results from mass spectrometry-based screening for interaction partners of FLAG-purified FBXL6. Log2FC values of co‐immunoprecipitated proteins in FBXL6 versus control samples are plotted against the *p*-value. FBXL6 (bait) is depicted in red and identified proteases in the interactome are marked with blue triangles. **I** FLAG-IP of FLAG-tagged full-length FBXL6 and FBXL6∆N, a fragment starting at Val48 to represent the cleaved form of FBXL6, with subsequent immunoblot analysis. **J** In-vitro-cleavage assay of THP-1 WCE incubated with specific inhibitors for Cathepsin G (CatG) and neutrophil elastase (NE) at increasing concentrations (2.5 µM, 25 µM or 250 µM) in addition to the standard inhibitor cocktail (Std. inhib.). Inc, incubation. **K** Immunoblot analysis of WCE prepared under standard (Std. lysis buffer) or denaturing conditions (SDS buffer) from MV4-11 or THP-1 pellets of equal size. Where indicated, excessive amounts of CatG-specific inhibitor were added to the standard lysis buffer.

When proceeding with the functional characterization of FBXL6, immunoblot analyses revealed a second form of FBXL6 running at a lower molecular weight (MW) of approximately 54 kDa (FBXL6-low_MW) next to the wild type (WT) form at the predicted molecular weight of 59 kDa (FBXL6-WT) (Fig. 2A, B). Importantly, FBXL6-low_MW was identified exclusively in AML cell lines, particularly of the M4 and M5 subtypes (according to the FAB classification) and not any other of the investigated hematological or solid cancer entities (Fig. 2A, B). FBXL6-low_MW was also found in AML patient-derived xenograft (PDX) cells (Fig. 2C). Knockdown experiments confirmed the specificity of this additional band (Supplementary Fig. 2A). Strikingly, FBXL6-low_MW was depleted upon TPA-induced myeloid differentiation while FBXL6-WT was enriched (Fig. 2D, Supplementary Fig. 2B–D). This shift to FBXL6-WT was also observed upon ATRA-induced differentiation (Supplementary Fig. 2E), suggesting a potential role of FBXL6-low_MW in maintaining the undifferentiated state in AML blasts. As no described isoforms of FBXL6 match the molecular weight of FBXL6-low_MW, we speculated that the lower form could result from proteolytic cleavage. To test this hypothesis, we cloned N- or C-terminally FLAG-tagged FBXL6 constructs and expressed them in AML cells. Interestingly, FBXL6-low_MW was only detectable by anti-FLAG antibodies upon C-terminal and not N-terminal tagging (Fig. 2E). This finding was strongly suggestive of proteolytic cleavage in the N-terminal region of FBXL6, resulting in a small N-terminal fragment not detectable in SDS-PAGE (Fig. 2E). Next, we performed in-vitro-cleavage assays based on prolonged incubation of lysates in presence of standard protease inhibitors or an expanded inhibitor cocktail additionally containing the protease inhibitors AEBSF, bestatin, E-64 and pepstatin. Reduced cleavage upon treatment with the expanded inhibitor cocktail further confirmed proteolytic processing of FBXL6 as the source for FBXL6-low_MW (Fig. 2F). Separate testing of the inhibitors identified AEBSF, an irreversible serine protease inhibitor, as the only inhibitor abrogating FBXL6-processing in vitro (Supplementary Fig. 3A, B), pointing toward cleavage by a protease of the serine-type. To identify the exact cleavage site, a dual proteomic approach combining top-down and bottom-up proteomics was applied (Supplementary Fig. 4A). The bottom-up approach, which measures the molecular weight of peptides resulting from trypsin digest, revealed the loss of peptides among amino acid (aa) 21 and aa 58 in the samples corresponding to FBXL6-low_MW (Supplementary Fig. 4B, C). Top-down proteomics, which determines the mass of the intact protein, resulted in the detection of three different fragments corresponding almost perfectly to the calculated molecular weight of cleavage products starting at Val48, Leu49 and Ser50 of FBXL6, thus mapping the cleavage site to aa 47-50 (Fig. 2G, Supplementary Fig. 4D, E). In order to identify the responsible protease, mass spectrometry-based interactome screening for FBXL6 interaction partners was performed next (Fig. 2H). Cross-validation of the FBXL6 interactome with a list of serine-proteases with a similar cleavage motif extracted from the MEROPS database [13] revealed cathepsin G (CatG) as the most promising candidate (Supplementary Fig. 5A). Indeed, CatG was found to interact with FBXL6-WT but not with FBXL6∆N, a mutant of FBXL6 lacking the first 47 aa to represent FBXL6-low_MW (Fig. 2I). Notably, a CatG-specific inhibitor efficiently abrogated in vitro FBXL6-processing, while an inhibitor for neutrophil elastase (NE), another myeloid serine-protease, had no effect (Fig. 2J). Moreover, we found that FBXL6 cleavage correlated with CatG expression across the cell line and PDX panels described in Fig. 2A–C (Supplementary Fig. 5B–D). CatG levels also correlated with changes in FBXL6 cleavage upon TPA or ATRA-induced differentiation (Supplementary Fig. 5E, F), confirming CatG as the responsible protease. Literature interrogation on CatG however made us aware of selected observations, in which aberrant proteolytic activity of CatG towards specific substrates was described as an artifact in standard lysis buffers without having a correlate in living cells [14, 15]. We therefore tested FBXL6 cleavage under denaturing conditions or in standard lysis buffer supplemented with excessive amounts of CatG inhibitor. Unexpectedly, cleaved FBXL6 was completely absent under these conditions (Fig. 2K), arguing against a physiological existence of cleaved FBXL6.

Taken together, we here nominate the ubiquitin ligase FBXL6 as a potential novel vulnerability in AML, thus providing further evidence for the important role of PTMs and specifically the ubiquitin system as a potential therapeutic target in AML. Moreover, we create awareness towards specific cleavage phenomena simulating biological relevance but ultimately representing artifacts due to high protease activity which is not sufficiently covered by standard protease inhibitor cocktails. This appears to be an experimental pitfall specifically in lysates of myeloid cells. Knowledge of these phenomena helps to plan according experiments under special precautions, including appropriate inhibitors and controls to prevent such artifacts.

### Supplementary information

Supplementary Information

Supplementary Table 1

## Data Availability

The mass spectrometry proteomics data have been deposited in the ProteomeXchange Consortium via the PRIDE partner repository with the dataset identifier PXD051269. Further datasets generated during the current study are included in this publication or are available from the corresponding authors on reasonable request.
