# Supplementary material for: FBXL6 is a vulnerability in AML and unmasks proteolytic cleavage as a major experimental pitfall in myeloid cells
Source: Leukemia. 2024 Jul 16;38(9):2027–31. doi: 10.1038/s41375-024-02345-0 (PMC11347359; doi:10.1038/s41375-024-02345-0)
Supplement: Supplementary file 1 — Supplementary Information [file 41375_2024_2345_MOESM1_ESM.pdf]

Supplementary information for

FBXL6 is a vulnerability in AML and unmasks proteolytic cleavage as a major experimental pitfall in myeloid cells

Anna Sperk[1,2], Antje Gabriel[1,2], Daniela Koch[1,2], Abirami Augsburger[1,2], Victoria Sanchez[3], David Brockelt[1,2], Rupert Öllinger[2,4], Thomas Engleitner[2,5], Piero Giansanti[2,6], Romina Ludwig[7], Priska Auf der Maur[1,2], Wencke Walter[8], Torsten Haferlach[8], Irmela Jeremias[7,9], Roland Rad[2,4,5], Barbara Steigenberger[3], Bernhard Kuster[10,11,12], Ruth Eichner[1,2*], and Florian Bassermann[1,2,11,12*]

*Corresponding authors.

Florian Bassermann (florian.bassermann@tum.de)

Ruth Eichner (ruth.eichner@tum.de)

Contents:

Supplementary Methods

Supplementary References

Supplementary Figures 1-5

Supplementary Methods

Culture of eukaryotic cells

All cell lines were grown in a humidified incubator at 37°C with 5% CO2 and cultured according to the supplier's recommendations (origin of cell lines and media composition is listed in the table below). All media were supplemented with 1% penicillin/streptomycin (Gibco). Heat inactivation (h.i.) of serum (FBS) was achieved by incubation for 60 min at 65°C. Adherent cells were kept on dishes and sub-cultured at 70-80% confluency. Suspension cells were grown in appropriately sized cell culture flasks at maximum densities between 1-10x10^5 cells/ml and split every 2-3 days at a ratio of 1:4-1:10. Cell numbers were determined using trypan blue exclusion counting in Neubauer chambers. All cells tested mycoplasma negative by a PCR detection method.

Cell line	Type (human)	Supplier	Medium
MOLM-13	AML	DSMZ (ACC 554)	RPMI + 10% FBS (h.i.)
OCI-AML3	AML	DSMZ (ACC 582)	AlphaMEM + 20%FBS (h.i.)
MV4-11	AML	DSMZ (ACC 102)	IMDM + 10% FBS (h.i.)
THP-1	AML	DSMZ (ACC 16)	RPMI + 10% FBS (h.i.)
HL-60	AML	DSMZ (ACC 3)	RPMI + 10% FBS (h.i.)
Kasumi-1	AML	DSMZ (ACC 220)	RPMI + 10% FBS (h.i.)
NB-4	AML	DSMZ (ACC 207)	RPMI + 10% FBS (h.i.)
PLB-985	AML	kind gift of Prof. P.Jost	RPMI + 10% FBS (h.i.)
NOMO-1	AML	DSMZ (ACC 542)	RPMI + 10% FBS (h.i.)
HEL	AML	DSMZ (ACC 11)	RPMI + 10% FBS (h.i.)
KG1a	AML	DSMZ (ACC 421)	RPMI + 10% FBS (h.i.)
K562	CML	DSMZ (ACC 10)	RPMI + 10% FBS (h.i.)
KCL-22	CML	DSMZ (ACC 519)	RPMI + 10% FBS (h.i.)
LAMA-84	CML	DSMZ (ACC 168)	RPMI + 10% FBS (h.i.)
HEK293T	embryonic kidney	ATCC (CRL-3216)	DMEM + 10% NCS
HeLa	cervival cancer	DSMZ (ACC 57)	DMEM + 10% FBS
U2OS	Osteosarcoma	ATCC (HTB-96)	McCoy's + 10% FBS (h.i.)

H1437	LuAD	ATCC (CRL-5872)	RPMI + 10% FBS (h.i.)
HCC44	LuAD	DSMZ (ACC 534)	RPMI + 10% FBS (h.i.)
MM1.S	MM	ATCC (CRL-2974)	RPMI + 10% FBS (h.i.)
L363	MM	DSMZ (ACC-49)	RPMI + 10% FBS (h.i.)
Amo1	MM	DSMZ (ACC-538)	RPMI + 10% FBS (h.i.)
Riva	DLBCL	DSMZ (ACC 585)	RPMI + 20% FBS (h.i.)
TMD8	DLBCL	kind gift of Prof. D. Krappmann	RPMI + 10% FBS (h.i.)

PDX samples

Cell pellets from from AML patient-derived xenograft cells (PDX) were provided by Irmela Jeremias and Romina Ludwig. The PDX samples were generated as previously described [1]. The table main genetic aberrations of the PDX samples are specified in the table below.

PDX Sample	Leading genetic abnormality
AML-372	monosomy 17, monosmy 7, ETV deletion, ATM deletion, NRAS, KRAS, TP53
AML-388	MLL-Rearrangement, KRAS
AML-393	MLL-Rearrangement, KRAS, BCOR
AML-491	DNMT3A, RUNX1, BCOR, PTPN11, KRAS, NRAS, ETV6, 7q31 deletion
AML-640	FLT3-ITD, NPM1, IDH1/2
AML-661	EZH2, BCOR, DNMT3A, ETV6, RUNX1, NRAS, PTPN11
AML-669	MLL-Rearrangement, KRAS

Plasmids, sgRNAs and shRNAs

To induce sgRNA-mediated knockout of *FBXL6* in Cas9-expressing (Addgene #52962 [2]) cells, the following sgRNA sequences were cloned into lentiGuide-GFP (modified from Addgene #52963 [2]): sgFBXL6_1 (5′-GGCTTATGCCCAATCGGTGA-3′), sgFBXL6_5 (5′-GAGCGTCAGCAGTCACACCG-3′), sgFBXL6_6 (5′-CAAGAAGCTCACCACAGCTG-3′), sgFBXL6_8 (5′-AGACCGGCTGACTCTAGCCA-3′), non-targeting sgRNA (5′-ACGGAGGCTAAGCGTCGCAA-3′), RNA polymerase II-targeting sgRNA (5'-CAACAAGATCACGCACGAAG-3').

For shRNA-mediated silencing, shRNAs were cloned into pLKO.1 TRC-DsRed2 (modified from Addgene #10878 [3]): shFBXL6_1 (5′-GCACCGGCATCAACCGTAATA-3′), shFBXL6_2 (5′-

AGACCGCATTCCCTTGGAAAT-3′), shFBXL6_3 (5′-CACCGGCATCAACCGTAATAG-3′), and shCtrl (5′-CCTAAGGTTAAGTCGCCCTCG -3′)

For ectopic expression of *FBXL6,* cDNA of *FBXL6* isoform 1 was cloned into pHIV-DsRed2 (modified from Addgene #21373 [4]) and pTRIPZ (modified from Thermo Fisher Scientific), with either N- oder C-terminal FLAG-tag. The fragment mutant starting at Val48 representing the cleaved form of *FBXL6* was cloned accordingly with a C-terminal FLAG-tag.

CRISPR/Cas9 drop-out screen

The F-box-focused CRISPR library was designed to target all 72 genes encoding F-box proteins in the human genome, together with essential genes as positive controls or non-targeting sgRNAs as negative controls. Three sgRNAs per gene were selected from the genome-wide human CRISPR-knockout library (GeCKOv2, [2]) and cloned into the lentiGuide-eGFP vector. Lentiviral particles of the pooled library were produced and titrated, and Cas9-expressing OCI-AML3 and MOLM-13 cells were lentivirally transduced at an MOI (multiplicity of infection) of 0.3 and an sgRNA coverage of approximately 2000. Successfully transduced cells were isolated as GFP positive by FACS using a FACSAria (BD Biosciences). Half of the resulting cells was harvested as day 0 sample, while the remaining cells were cultured for 14 days before harvesting. Genomic DNA was isolated, sgRNA cassettes were amplified by PCR to add barcodes for next-generation sequencing (NGS). Sequencing was carried out on a MiSeq Illumina machine using the MiSeq Reagent Kit v2 (Illumina). Reads were aligned to the sgRNA sequence library and read counts were determined for each sgRNA. After normalization to the total number of reads, enrichments and dropouts were calculated between day 0 and day 14 samples.

Flow cytometry

Data presented in this study were either obtained at a FACS Calibur or a FACS Accuri C6 plus (both BD Biosciences) and analyzed using the software FlowJo v10. To determine the transduction efficiency of AML cell lines infected with a lentiviral vector containing GFP as a fluorescent marker, cells from the growing culture were collected at the indicated time points, washed with PBS and analyzed for GFP-positivity using flow cytometry.

MTS cell viability assay

To estimate the number of viable, metabolically active cells, AML cells were subjected to MTS assays by plating 100 µl of homogenous cell suspension into flat-bottom 96-well plates and adding 20 µl of CellTiter 96® Aqueous One Solution (Promega). After 1-2 hrs incubation at 37°C, absorbance at 490 nm was measured on a GloMax Explorer Multimode Microplate Reader (Promega). Absorbance values were reduced by the background signal determined from medium controls and depicted in relation to an internal control.

Production of lentiviral particles and viral transduction of cells

Production of lentiviral particles and viral transduction was performed as described previously [5, 6]. In brief, HEK293T cells were transiently transfected in a 10 cm dish using the calcium phosphate method to deliver 15 µg packaging plasmid (psPAX2, Addgene #12260, D. Trono), 5 µg envelope plasmid (pMD2.G, Addgene #12259, D. Trono) and 20 µg of the plasmid of interest (e.g. an sgRNA construct or a pHIV-based overexpression construct). 24 hrs after transfection, the medium was replaced with 6-10 ml Opti-MEM, which was harvested as viral supernatant after another 24 hrs. To clear the supernatant from cell debris, it was passed through a 0.45 µm filter and used either directly or stored at -80°C.

For lentiviral infection of AML cell lines, 0.5-1x10^6 cells were plated in 0.5 ml of growth medium per well of a 6-well plate. 2 ml of viral supernatant and polybrene at a final concentration of 8 µg/ml were added. The cells were subjected to spin-infection at 700xg for 30 min at 30°C and subsequently incubated with the viral supernatant for 24 hrs before exchanging the medium.

Preparation of whole-cell extracts and *in-vitro*-cleavage assays

The standard lysis buffer consisted of 50 mM TRIS pH 7.5, 150 mM NaCl, 0.1% NP-40, 5 mM EDTA, 5 mM $MgCl_2$, 5% glycerol supplemented with 1 mM DTT, protease inhibitors (0.1 mM PMSF, 5 µg/ml TLCK, 10 µg/ml TPCK, 1 µg/ml aprotonin, 1 µg/ml leupeptin, 10 µg/ml soybean trypsin inhibitor), phosphatase inhibitors (0.1 mM Na_3VO_4, 10 mM Glycerol-2-Phospate) and benzonase (1:3,000). The expanded inhibitor cocktail additionally comprised 0.5 mM AEBSF, 20 µM bestatin, 10 µM E-64, and 20 µM pepstatin. For lysis, cell pellets were resuspended in ice-cold lysis buffer and pressed through a syringe for organelle break-up. Cleared lysates were either used for immunoprecipitations or *in-vitro*-cleavage assays or were denatured by the addition of Laemmli buffer and heating to 95°C for 5 min.

For denaturing lysis, cell pellets were boiled in 2% SDS-containing lysis buffer (10 mM TRIS pH 8.5). Lysates were subjected to one freeze-thaw cycle (-80°C, followed by boiling for 5 min 95°C) and acidified with 10% TFA (Trifluoroacetic acid, final concentration 1-2%), vortexed and quenched with 20% NMM (4-Methylmorpholine, final concentration 2-4%).

Immunoblotting

Immunoblotting was performed as described previously [5-7]. Proteins separated by SDS-PAGE were transferred to methanol-activated polyvinylidene difluoride (PVDF) membranes (Merck Millipore) by electroblotting. After blocking in 5% milk for 30 min, the membranes were incubated with primary antibodies (see table below) diluted in 5% milk or 5% BSA at 4°C overnight. After washing three times in wash buffer, membranes were incubated with the respective HRP (horseradish peroxidase)-coupled secondary antibody (see table below) at a dilution of 1:15,000 in 5% milk for 45 min at RT. After washing again three times, blots were incubated with enhanced chemiluminescent (ECL) solution (Pierce™ ECL Western Blotting Substrate, Thermo Fisher Scientific) and exposed to photosensitive films (Amersham HyperfilmT[M] ECL). An automatic film developer machine (Curix 60, Agfa) was used to visualize protein bands.

Antibody (clone)	Dilution (application)	Supplier (catalog#)
Cathepsin G (clone 12H15L69)	1:1000 (IB)	Thermo Fisher Scientific (#703590)
CUL1	1:1000 (IB)	Abcam (#ab85152)
ECL anti-mouse IgG, HRP-linked	1:15 000 (IB)	GE Healthcare (#NA931)
ECL anti-rabbit IgG, HRP-linked	1:15 000 (IB)	GE Healthcare (#NA934)
Erk1/2 (clone C-9)	1:1000 (IB)	Santa Cruz (#sc-514302)
FBXL6	1:400 (IB)	Thermo Fisher (#PA564927)
GAPDH	1:1000 (IB)	Santa Cruz (#sc-47724)
p27	1:1000 (IB)	BD Pharmingen (#554069)
Phospho-Erk1/2 (Thr202/Tyr204)	1:1000 (IB)	Cell Signaling (#9101S)
β-Actin	1: 3000 (IB)	Sigma (#A2228)

Immunoprecipitation and mass spectrometry

FLAG-FBXL6 was purified using FLAG-M2 beads (Sigma) and eluted with 1 mg/ml 3XFLAG peptide from AML cells. Samples for bottom-up proteomics (cleavage site identification) were separated via SDS-PAGE and stained with Coomassie. Bands corresponding to FBXL6 were isolated and subjected to trypsin digest for peptide identification. A fraction of the samples was used for direct injection into the mass spectrometer to determine the total protein mass of cleaved and full-length FBXL6 (top-down proteomics). For bottom-up proteomics, tryptic peptides were extracted, dried, reconstituted in buffer containing 0.1% formic acid (FA) and analyzed by LC-MS/MS (liquid chromatography tandem mass spectrometry) on a 30-cm column (inner diameter: 75 microns; packed with ReproSil-Pur C18-AQ 1.9-micron beads, Dr. Maisch GmbH) coupled to an Exploris 480 mass spectrometer (Thermo Fisher Scientific) for the cleavage site identification. Peptide and protein identification and quantification were performed using MaxQuant. For top-down proteomics, samples were measured on an Agilent 1100 HPLC column (Phenomenex Aeris™ 3.6 μm WIDEPORE C4) coupled to a microTOF mass spectrometer (Bruker Daltonics, mode: positive, mass range 800-3000 m/z). Total mass data was analyzed the complementary Bruker Data analysis software.

For interaction proteomics, FLAG-FBXL6 was purified as described above and separated via SDS-PAGE. Digests of each band were loaded for 10 min (0.1% FA, 5 µL/min) on the trap column, peptides were transferred to the analytical column and separated at 300 nL/min using a 8 min 2-steps gradient from 4% to 24% to 36% LC solvent B (0.1% FA, 5% dimethyl sulfoxide (DMSO) in acetonitrile) in LC solvent A (0.1% FA in 5% DMSO). To improve chromatographic separation and reduce dead time, the flow was maintained at 500 nL/min for the first 5 min. Total analysis time was 15 min. The Orbitrap Eclipse was operated in data dependent and positive ionization mode. MS1 spectra were recorded in the Orbitrap from 360 to 1300 m/z at a resolution of 60K (automatic gain control (AGC) target value of 100%, maximum injection time (maxIT) of 50 ms). Peptide fragmentation was performed via higher energy collisional dissociation (normalized collision energy of 30%), and MS2 spectra were recorded in the Orbitrap at 30K resolution via sequential isolation of the most abundant precursors within the selected cycle time of 0.9 s (isolation window 1.3 m/z, AGC target value of 100%, maxIT of 54 ms, and dynamic exclusion of 25 s).

Raw mass spectrometry data were processed using the MaxQuant software (version 2.0.3.0) with its built-in search engine, Andromeda [8]. Spectra were searched against the UniProtKB database (Human,

UP000005640, 79,759 entries downloaded on 09.2022). Enzyme specificity was set to trypsin, allowing for 2 missed cleavages, and the search included cysteine carbamidomethylation as a fixed modification, and protein N-term acetylation and methionine oxidation as variable modifications. Identifications were adjusted to 1% false discovery rate (FDR) at protein and peptide levels. The match-in-between runs and the second peptide options were enabled. The MaxLFQ algorithm was used for Label Free Quantification (LFQ).

Data analysis was performed using the Perseus software (version 2.0.7.0.). Protein identifications were filtered to remove contaminants and decoy, and LFQ intensity values were log2-transformed. For statistical analysis, only proteins that had been quantified in at least 2 of the 3 biological replicates in at least one sample type (empty vector or bait FLAG-FBXL6 IP) were retained, and missing values were imputed via the normal distribution algorithm implemented in Perseus, using default parameters. Significance was assessed by a two-tailed Welch's t-test comparing the FLAG-FBXL6 IP and the empty vector control. Multiple hypothesis testing was controlled by using a Benjamini-Hochberg FDR threshold of 5%.

The mass spectrometry proteomics data have been deposited in the ProteomeXchange Consortium via the PRIDE partner repository [9] with the dataset identifier PXD051269 (Website: http://www.ebi.ac.uk/pride, Username: reviewer_pxd051269@ebi.ac.uk, Password: 6ByLx9kx).

Statistical Analysis

All quantified experiments were performed in triplicates. The non-quantified immunoblot data generally shows results representative of at least two independent experiments. Statistical analyses of the results were performed with the GraphPad Prism v9 software. Depending on the type of data, significance was calculated using the Student's t-test or one-sample t-test, according to assumptions of the test. Statistical analysis of relative ratios was performed using one-sample t-tests with hypothetical means of 1.0. The error bars shown in the figures represent the mean ± standard deviation (S.D.). The P values are denoted in the figure legends where a statistically significant difference was found: *, $P < 0.05$; **, $P < 0.01$; ***, $P < 0.001$; ****, $P < 0.0001$.

Supplementary References

1. Vick B, Rothenberg M, Sandhofer N, Carlet M, Finkenzeller C, Krupka C, et al. An advanced preclinical mouse model for acute myeloid leukemia using patients' cells of various genetic subgroups and in vivo bioluminescence imaging. PLoS One. 2015;10(3):e0120925. doi: 10.1371/journal.pone.0120925.

2. Sanjana NE, Shalem O, Zhang F. Improved vectors and genome-wide libraries for CRISPR screening. Nat Methods. 2014;11(8):783-4. doi: 10.1038/nmeth.3047.

3. Moffat J, Grueneberg DA, Yang X, Kim SY, Kloepfer AM, Hinkle G, et al. A lentiviral RNAi library for human and mouse genes applied to an arrayed viral high-content screen. Cell. 2006;124(6):1283-98. doi: 10.1016/j.cell.2006.01.040.

4. Welm BE, Dijkgraaf GJ, Bledau AS, Welm AL, Werb Z. Lentiviral transduction of mammary stem cells for analysis of gene function during development and cancer. Cell Stem Cell. 2008;2(1):90-102. doi: 10.1016/j.stem.2007.10.002.

5. Heider M, Eichner R, Stroh J, Morath V, Kuisl A, Zecha J, et al. The IMiD target CRBN determines HSP90 activity toward transmembrane proteins essential in multiple myeloma. Mol Cell. 2021;81(6):1170-86 e10. doi: 10.1016/j.molcel.2020.12.046.

6. Paulmann C, Spallek R, Karpiuk O, Heider M, Schaffer I, Zecha J, et al. The OTUD6B-LIN28B-MYC axis determines the proliferative state in multiple myeloma. EMBO J. 2022;41(20):e110871. doi: 10.15252/embj.2022110871.

7. Eichner R, Heider M, Fernandez-Saiz V, van Bebber F, Garz AK, Lemeer S, et al. Immunomodulatory drugs disrupt the cereblon-CD147-MCT1 axis to exert antitumor activity and teratogenicity. Nat Med. 2016;22(7):735-43. doi: 10.1038/nm.4128.

8. Cox J, Mann M. MaxQuant enables high peptide identification rates, individualized p.p.b.-range mass accuracies and proteome-wide protein quantification. Nat Biotechnol. 2008;26(12):1367-72. doi: 10.1038/nbt.1511.

9. Perez-Riverol Y, Bai J, Bandla C, Garcia-Seisdedos D, Hewapathirana S, Kamatchinathan S, et al. The PRIDE database resources in 2022: a hub for mass spectrometry-based proteomics evidences. Nucleic Acids Res. 2022;50(D1):D543-D52. doi: 10.1093/nar/gkab1038.

Supplementary Figures:

Supplementary Fig. 1

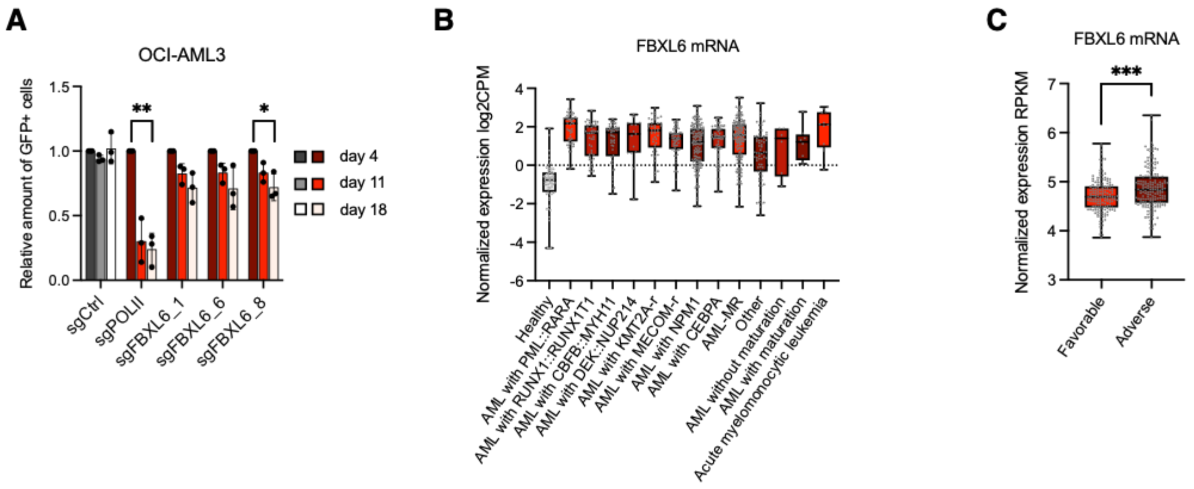

Supplementary Fig. 1 FBXL6 is a vulnerability in AML. A Competitive growth assay of *Cas9*-expressing OCI-AML3 cells transduced with GFP-expressing sgRNA constructs targeting *FBXL6* (sgFBXL6), *POLII* (sgPOLII) as positive control or non-targeting control (sgCtrl) at 30-50% efficiency. The ratio of GFP-positive to non-transduced cells was measured by flow cytometry on the indicated days and normalized to day 2. ***; P < 0.001; **, P < 0.01; *, P < 0.05, by One sample t-test. **B** Individual values of *FBXL6* mRNA expression in the AML patient cohort described in Fig. 1F and G by specific AML subtype. Classification according to the current WHO guidelines, only subtypes with at least 6 cases are shown. CPM, counts per million reads mapped. **C** Individual *FBXL6* mRNA expression values from the patients of the BeatAML2.0 cohort, subdivided into favorable and adverse risk classified according to the European LeukemiaNet (ELN-2017) guidelines. RPKM, reads per kilobase of transcript per million reads mapped. ***, P < 0.001, by Student's t-test.

Supplementary Fig. 2

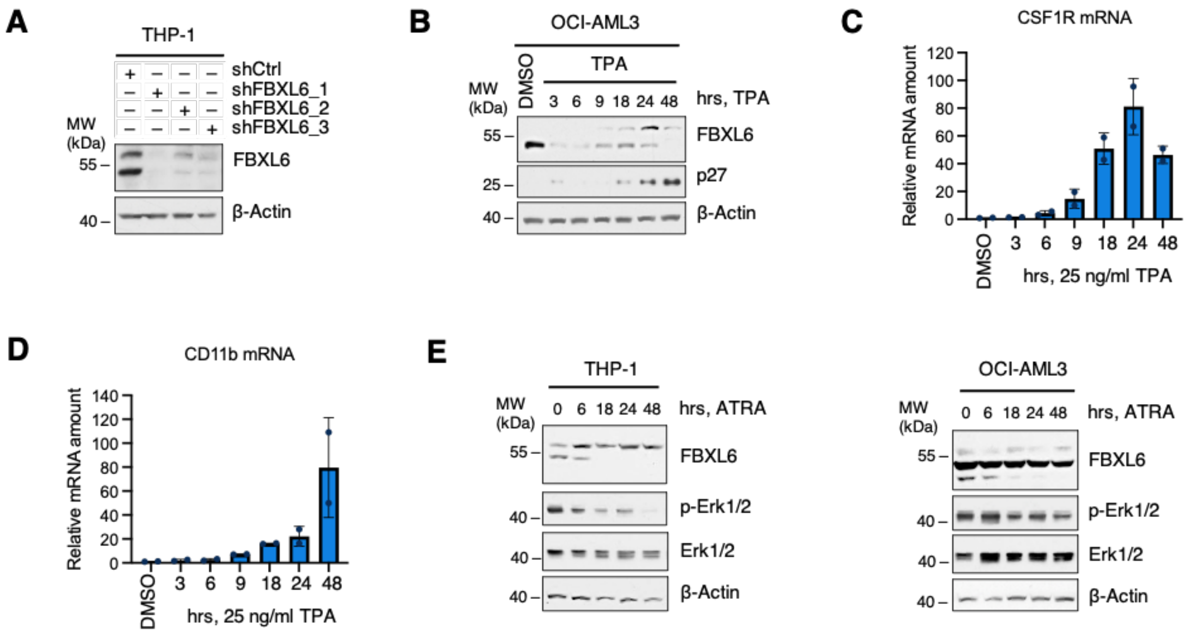

Supplementary Fig. 2 The lower molecular weight form of FBXL6 is specific to undifferentiated AML cells. A Immunoblot analysis of THP-1 cells transduced with shRNA constructs targeting *FBXL6* or non-targeting control (shCtrl). Cells were harvested 3 days after infection and subjected to cell lysis in standard conditions with subsequent SDS-PAGE and immunoblot analysis. **B** Representative Immunoblot analysis of OCI-AML3 cells treated with 25 ng/ml TPA or DMSO control for the indicated periods of time. WCE were prepared under standard lysis conditions . **C** Relative quantification of *CSF1R* mRNA levels as indicator of myeloid differentiation from samples in (B) and two additional biological replicates. Values are normalized to RPLP0 and shown in relation to DMSO control. **D** Quantification of *CD11b* mRNA levels as indicator of differentiation in THP1 samples shown in Fig. 2D and two additional biological replicates. Values are normalized to RPLP0 and shown in relation to DMSO control. **E** Representative immunoblot analyses of THP-1 and OCI-AML3 cells treated with 1 μM ATRA for the indicated periods of time. Whole-cell extracts were prepared under standard lysis conditions.

Supplementary Fig. 3

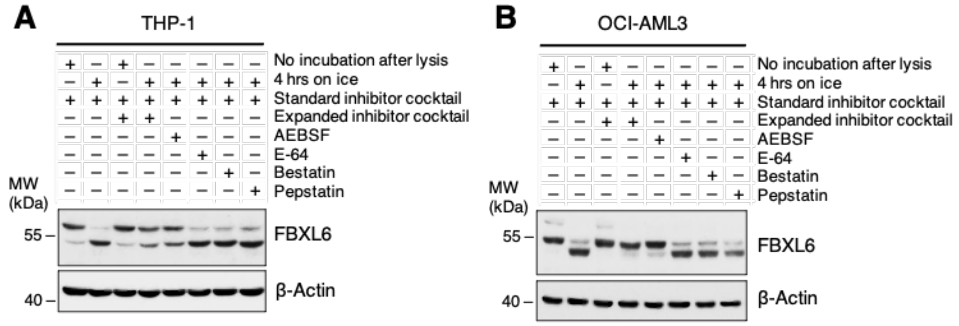

Supplementary Fig. 3. FBXL6 is cleaved in AML cells by a protease of the serine-type. A-B Representative Immunoblot analyses of *in-vitro*-cleavage assays under different conditions. THP-1 (A) or OCI-AML3 (B) cell lysates were incubated on ice or at 25°C for the indicated periods of time or denatured directly after lysis by addition of Laemmli buffer. Standard inhibitor cocktail contains aprotinin, leupeptin, soybean trypsin inhibitor, PMSF, TPCK, TLCK; expanded inhibitor cocktail additionally comprises AEBSF, bestatin, E-64, and pepstatin.

Supplementary Fig. 4

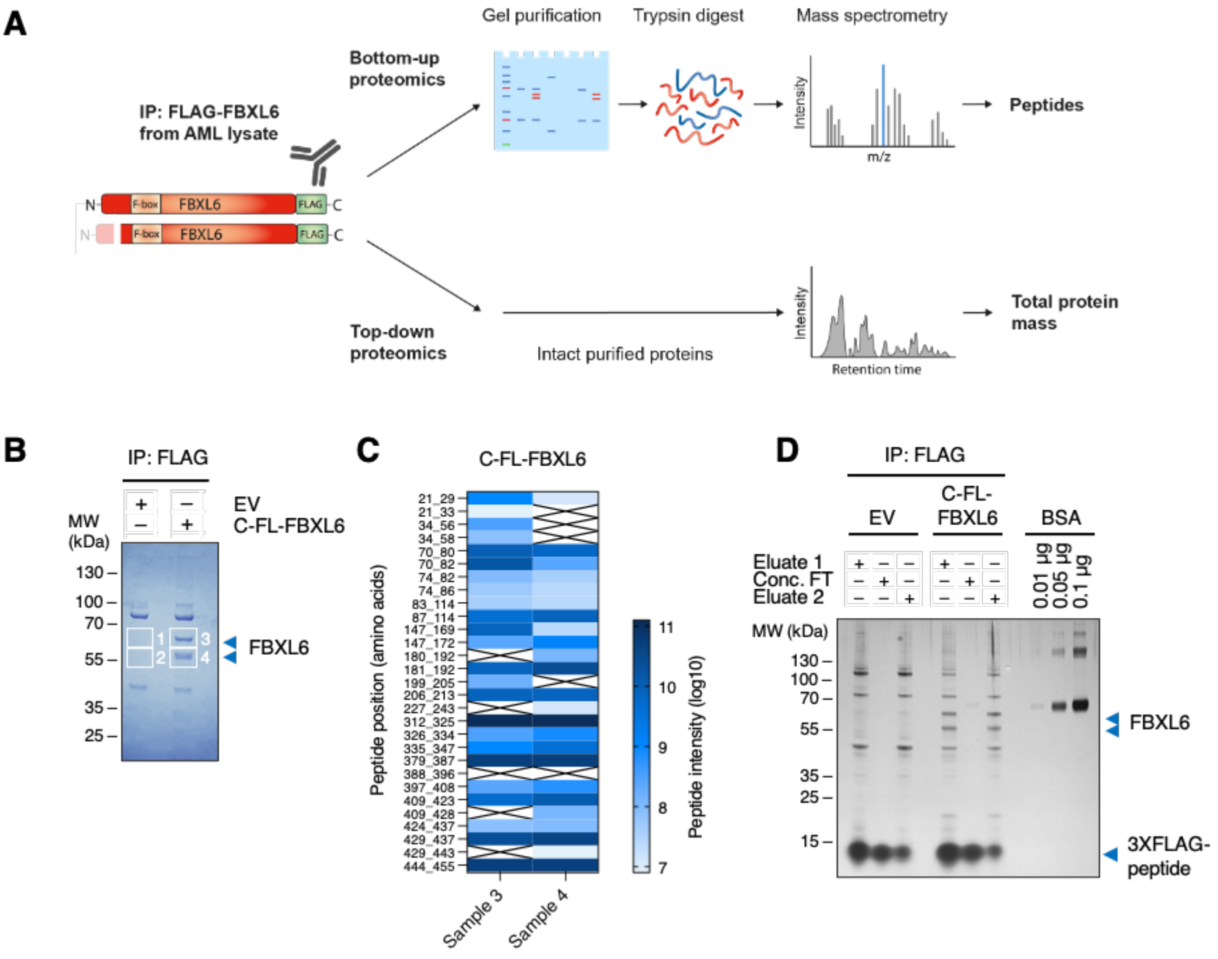

E

Begin of C-term. fragment	Val (48)	Leu (49)	Ser (50)
calculated MW (Da)	54 153.87	54 054.73	53 941.57
measured MW (Da)	54 155.36	54 032.32	53 941.09

Supplementary Fig. 4 FBXL6 is cleaved between Leu47 and Ser50. A Schematic of the dual mass spectrometry-based approach to identify the cleavage site of FBXL6. C-terminally FLAG-tagged FBXL6 (C-FL-FBXL6) versus empty vector (EV) control was purified from THP-1 cells via immunoprecipitation (IP) using FLAG-M2 beads and eluted with 3XFLAG peptide. Half of the sample was subjected to trypsin digest for mass spectrometric peptide identification (bottom-up proteomics), while the other half was used for direct mass spectrometric measurement of total protein mass of FBXL6-low_MW versus full-length FBXL6 (top-down proteomics). **B** Coomassie staining of eluted proteins of samples described in (A). Rectangles mark the isolated gel slices for bottom-up proteomics (1 and 2 for EV control, 3 and 4 for FBXL6-WT and FBXL6-low_MW respectively). **C** Peptide intensities within sample 3 and 4

(described in B) as measured by mass spectrometry. Crossed out peptides mark peptides not detected in the respective samples. **D** Silver staining of the same FLAG-FBXL6 eluates described in (A) and (B), which were subjected to centrifugal protein concentration for top-down proteomics. Equivalent amounts of original eluate (Eluate 1), concentrator flow through (Conc. FT), and concentrated eluate (Eluate 2) were loaded. Bovine serum albumin (BSA) served as reference for purified protein amount. **E** Comparison of total protein masses determined by mass spectrometry (top-down proteomics) with calculated molecular weights based on the FBXL6 amino acid sequence. Values are derived from two biological replicates.

Supplementary Fig. 5

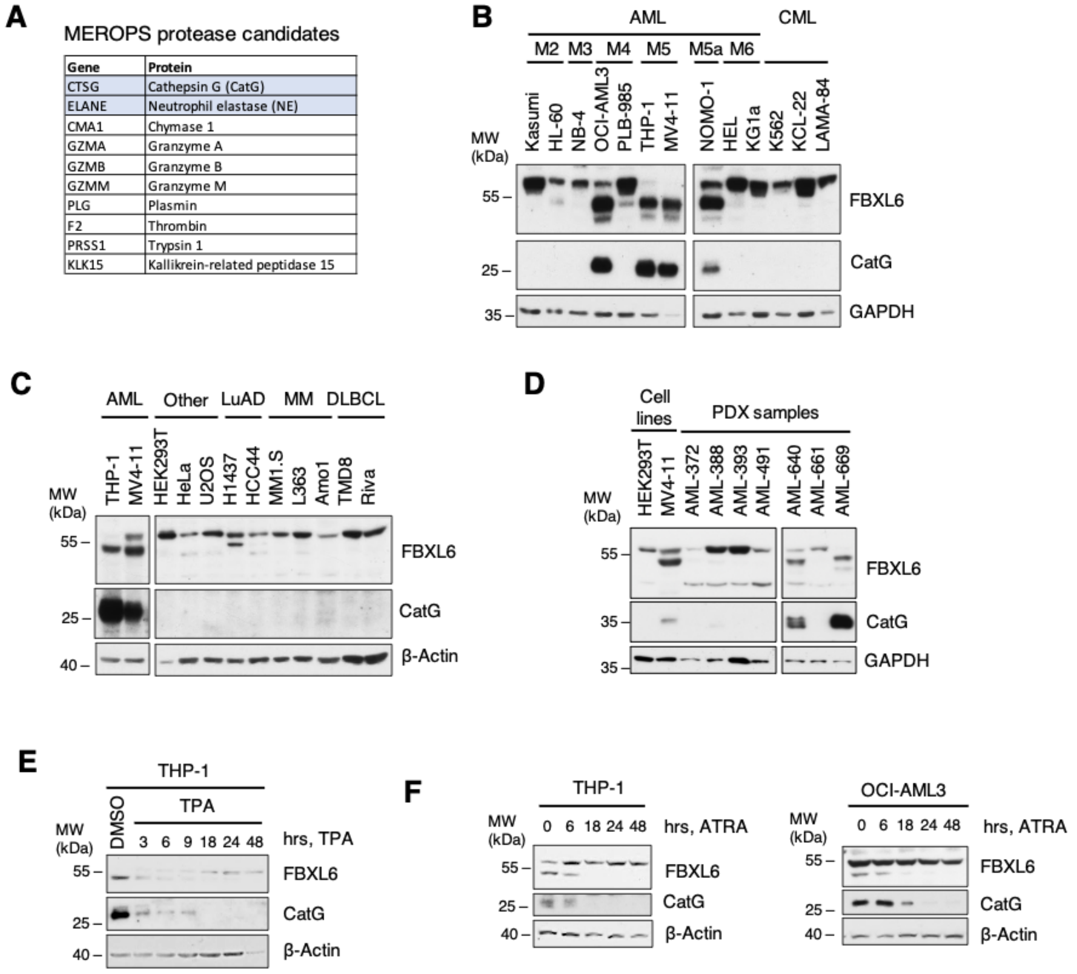

Supplementary Fig. 5. Identification of CatG as the FBXL6-directed protease. A Results from the MEROPS database (Rawlings et al., 2014) regarding proteases with a comparable target motif as identified for FBXL6. The resulting 32 enzymes were filtered for serin proteases. Proteases that are expressed in myeloid cells are marked in blue. **B-D** Immunoblot analyses of WCEs from various cell lines and PDX samples as described in Fig. 2A-C, here additionally probed with CatG antibody. **E-F** Immunoblot analyses of AML cells treated with 25 ng/ml TPA (**E**) or 1 μM ATRA (**F**), as shown in Fig. 2D and Supplementary Fig. 2E, here additionally probed with Cat G antibody.
